# Supplementary material for: A fungal metabolic regulator underlies infectious synergism during Candida albicans-Staphylococcus aureus intra-abdominal co-infection
Source: Nat Commun. 2024 Jul 9;15:5746. doi: 10.1038/s41467-024-50058-w (PMC11233573; doi:10.1038/s41467-024-50058-w)
Supplement: Supplementary file 1 — Supplementary Information [file 41467_2024_50058_MOESM1_ESM.pdf]

A fungal metabolic regulator underlies infectious synergism during *Candida albicans*-*Staphylococcus* aureus intra-abdominal co-infection

SUPPLEMENTARY METHODS

Phenotypic profiling of *C. albicans* strains

Growth was assessed by diluting overnight YPD grown cultures to 1x10^5 cells/mL in fresh YPD (or other media as specified), transferring 200 µL to a microtiter plate, and kinetically measuring the OD600 nm at hourly intervals using a BioTek Synergy spectrophotometer. In some experiments, the pH of culture supernatants was assessed by first filter sterilizing the spent medium using a 0.22 µm filter and using a standard laboratory pH meter. Additionally, hyphal growth assays were carried out by diluting (1:100) overnight cultures of YPD grown cells in Roswell Park Memorial Institute 1640 medium (RPMI) and incubating at 37ºC with shaking. Aliquots were removed at 2 and 4 h post-inoculation, transferred to glass slides, and images digitally captured by standard light microscopy. To determine susceptibility to common stressors YPD grown cells were washed 3X and diluted in sterile water. Aliquots containing 5-5x10^4 cells were spotted onto YPD plates containing the following stressors: 100 mM $CaCl_2$, 500 mM $CaCl_2$, 100 µg/mL Congo Red, 3% ethanol, 3% glycerol, 10 mM $MnCl_2$, 1.5 M NaCl, 0.05% SDS, 1 M sorbitol, 5 mM H_2O_2, 1 µg/mL fluconazole, or 5 µg/mL fluconazole. Susceptibility to pH was similarly tested in YPD medium adjusted to pH 3.5 or 8.5. Plates were incubated at 30ºC and growth assessed at 24 h. Temperature sensitivity was determined by plating onto YPD and incubating at 37ºC or 42ºC for 24 h. Images were acquired digitally using a Gel Doc XR Imaging System.

α-toxin ELISA protocol

Microtiter plates were coated with 50 µL 0.1 µg/mL anti-α-toxin antibody MEDI4893* diluted in ELISA/ELISPOT coating buffer (Invitrogen). Plates were incubated at 4°C overnight, washed with PBS-Tween 20 (PBS-T), and blocked with SuperBlock (Pierce, Cat No. 37515) for 1 h at room

temperature. Then, 50 µL filter-sterilized culture supernatant, peritoneal lavage fluid, or clarified organ homogenate were serially diluted in PBS and plated along with native α-toxin as the standard. Plates were incubated for 1 h at room temperature and then washed with PBS-T. Affinity-purified rabbit polyclonal anti-α-toxin antibody (2 µg/mL) was added for 1 h at room temperature. Plates were washed with PBS-T. AffiniPure horseradish peroxidase (HRP)-coupled goat anti-rabbit IgG detection antibody (Jackson ImmunoResearch, Cat. No. 111-035-003) was diluted 1:10000 and incubated for 1 h at room temperature. After extensive washing with PBS-T, 100 µL 3,3',5,5'-Tetramethylbenzidine (TMB) (Thermo Scientific, Cat. No. 34021) substrate was added, and plates were incubated in the dark for 10 min. 100 µL of 0.2 N H_2SO_4 was immediately added to stop the reaction. Plates were read at 450 nm using a BioTek Synergy H1. Experimental concentrations were extrapolated to the standard curve. Culture supernatants or in vivo samples from the *S. aureus hla*::*bursa* strain were blank subtracted to account for any nonspecific antibody binding to shed protein A.

Site-directed mutagenesis

Construction of the oligomerization-deficient H35L α-toxin mutant was performed using Platinum SuperFi I (Invitrogen, Cat. No. 12358010) following the manufacturer's site-directed mutagenesis protocol. Primers H35L-Superfi-F and H35L-Superfi-R **(Supplementary Table 3)** were used to PCR amplify previously constructed pSK-hla [1]. Amplified DNA was digested with DpnI to remove residual template DNA and transformed into the *dcm*-deficient *E. coli* strain IM08B to yield plasmid pSK-H35L (**Supplementary Table 4**). pSK-H35L was electroporated into *S. aureus* NE1345 *hla*::*bursa* and selected on TSA with 10 µg/mL chloramphenicol to create strain *hla*::*bursa*-p*hla*^{H35L}. Sanger sequencing using primers hlaSeqF and hlaSeqR was performed to verify plasmid integrity.

Construction of the *agr* P3 luciferase reporter

To construct a P3-luciferase reporter plasmid, the *S. aureus agr* P3 promoter was PCR amplified

from synthetic DNA (Integrated DNA Technologies [IDT]) using primers SynthGeneF and SynthGeneRv4 **(see Supplemental Table 5).** This PCR product and plasmid pMV306G13+Lux (Addgene, #26160) containing the *luxABCDE* operon were digested with NotI and NcoI, ligated, and transformed into *E.coli* DH5-α to yield pOLux1 using the method previously described [2]. The entire P3-*luxABCDE* fragment from pOLux1 was PCR amplified using primers luxABCDE-Fv2-EcoRI and luxABCDE-R-NarI. This product, along with plasmid pMK4, were digested with EcoRI and NarI. Digests were ligated and transformed into *E. coli* DH5-α, yielding plasmid pOLux2. The constitutive *S. aureus* promoter *sarA* P1 was synthesized (IDT) and amplified with primers SynthGeneF and SynthGeneRv4 (**Supplementary Table 5**). This product and pOLux2 were digested with Acc65I, ligated, and transformed into *E. coli* DH5-α, yielding pOLux3 (**Supplementary Table 4**). Correct orientation of the *sarA* P1 promoter in pOLux3 was confirmed by PCR amplification with primers sarAP1-FAcc65I and luxC-DET-R. pOLux3 was transformed into the *dcm*-deficient *E. coli* strain IM08B, as previously described [2]. After confirmatory digestion and sequence integrity verified, plasmids were transformed into *S. aureus* JE2 competent cells and plated on brain heart infusion agar containing 10 μg/mL chloramphenicol to yield strain *S. aureus*(pOLux).

***ZCF13* disruption plasmid construction**

Plasmids for *ZCF13* gene disruption were constructed using plasmid pBSS2, containing the *SAT1*-flipper disruption cassette [3]. The 5' flanking region of the *ZCF13* ORF was amplified from SC5314 genomic DNA using primers ZCF13-FF-KpnI and ZCF13FR-ApaI, digested with KpnI and ApaI, and ligated into pBSS2 at the KpnI and ApaI restriction sites to create pBSS-2ZCF13-F. The 3' flanking region of the *ZCF13* ORF was amplified from SC5314 gDNA using primers ZCF13-RF-NotI and ZCF13-RR-SacI, digested with NotI and SacI, and ligated into pBSS2-ZCF13-F at the NotI and SacI restriction sites to create pBSS2-ZCF13-FR (**Supplementary Table 4**).

***ZCF13* mutant construction**

Plasmid pBSS2-ZCF13-FR was digested with KpnI and SacI and the linear fragment was transformed into SC5314 using a standard lithium acetate protocol, with some modifications [4, 5]. An overnight culture of SC5314 was diluted 1:200 in 5 mL YPD and grown for 6 h at 30°C with shaking at 200 rpm and washed by centrifugation using sterile water. Cells were then resuspended in 1X TELiAc buffer (100 mM LiAc, 1X TE) and 1 µg of linearized pBSS2-ZCF13-FR was added to 50 µL cell suspension. Single-stranded salmon sperm carrier DNA (5 µL) was added along with 300 µL 40% PEG. The transformation mix was incubated at 30°C for 30 min with agitation every 10 min. Cells were heat-shocked at 42°C for 15 min and then allowed to recover in YPD at 30°C with shaking at 200 rpm for 4-6 hours. Cells were pelleted and resuspended in 1X TE and plated on YPD+200 µg/mL nourseothricin (YPD+200NAT). Plates were incubated for up to 2 days at 30°C. Colonies were grown overnight in 1 mL YPM (YP+2% maltose) to induce cassette excision. Cells from YPM cultures were washed with PBS and then diluted to 10^3 cells in 1X TE buffer. 100 µL cells were plated onto YPD+25 µg/mL nourseothricin (YPD+25NAT) and incubated at 30°C for 24 hours. Small and medium-sized colonies were patched onto YPD and YPD+200NAT and incubated at 30°C for 24 hours. Colonies that grew on YPD but not YPD+200NAT were confirmed by PCR to have *ZCF13* disrupted using primers ZCF13-FLPINTF + ZCF13-DET-R and ZCF13-FLPINTR + ZCF13-DET-F. The transformation procedure was repeated to disrupt the second *ZCF13* allele. Homozygous deletion of *ZCF13* was verified with primers ZCF13-DET-F + ZCF13-DET-R.

***ZCF13* revertant construction**

One copy of *ZCF13* was inserted into the neutral locus *NEUT5L* of *zcf13*Δ/Δ using the shuttle vector pDUP3. The *ZCF13* 5' UTR, ORF, and 3' UTR was PCR amplified from SC5314 using primers ZCF13-AMPF-SmaI and ZCF13-AMPR-NotI. The PCR product and plasmid pDUP3 were digested with SmaI and NotI and ligated to create pDUP3-ZCF13 (**Supplementary Table 4**). After transformation into DH5α and plasmid recovery, pDUP3-ZCF13 was linearized by digesting with

SfiI and then transformed into *zcf13*Δ/Δ following the lithium acetate protocol described above. Revertant colonies were selected for by growth on YPD+200NAT and confirmed by PCR using primers NAT1INTF + NEUT5LAMPF and NEUT5LAMPR + ZCF13-DET-R.

Construction of *rbk1*Δ/Δ

To construct the *rbk1*Δ/Δ strain, linearized *SAT1*-flipper and *CaHygB*-flipper plasmids (**Supplementary Table 4**) were amplified using Platinum™ II Taq Hot-Start DNA polymerase (Invitrogen, Cat. No. 14966100) with RBK1CC9KO-F and RBK1CC9KO-R primers to make the disruption repair templates harboring nourseothricin or hygromycin resistance cassettes, respectively as described [6, 7]. Before the transformation, *C. albicans* competent cells were treated with 1X Tris-EDTA and 0.1 M lithium acetate (pH 7.5) for 1 h at 37°C followed by addition of 25 mM dithiothreitol for another 30 minutes. Cells were then washed with sterile water and 1 M sorbitol and resuspended in residual sorbitol. The cells were mixed with premade ribonucleoprotein (RNP) complex containing universal tracrRNA (IDT, Cat. No. 1072532), 2 µg Cas9 protein (IDT, Cat No. 1081058), *RBK1* gene-specific guide RNAs (crRBK1up and RBK1down), and 1 µg of each PCR generated repair template. The transformation was performed by a single pulse at 1.8 kV using the Gene Pulser Xcell Electroporation System (Bio-Rad). Immediately, cells were incubated at 30°C for 4-6 hours in YPD medium and plated on YPD+200NAT and 600µg/mL hygromycin B (YPD+600HygB). Selected colonies were transferred to a yeast-peptone medium with 2% maltose (YPM) for overnight culture to excise the cassettes. Cells were then selected in YPD agar media with nourseothricin (25 µg/mL) and hygromycin (75µg/mL). Genetic integration and excision of the resistance cassettes were confirmed phenotypically by growth on YPD, YPD+200NAT, and YPD+600HygB solid media. Correct genomic integration and loss of *RBK1* was confirmed by PCR amplification of genomic DNA using primers RBK1INTF+FLPINTR, RBK1INTR+FLPINTF, and RBK1DETF+RBK1DETR **(Supplementary Table 3)** [6, 7].

Construction of the *rbk1*Δ/Δ+*RBK1* strain

To make the *rbk1*Δ/Δ+*RBK1* strain, part of the *NEUT5L* homology region, *RBK1* promoter and ORF, and ADH1terminator were PCR amplified from either SC5314 gDNA or pDIS3-tAHD1 plasmid using primer pairs NEUT5homology-pDISF+Nt5ADHup-UNIVOL-R, RBK1_Pr_OL-F+RBK1_ORF-OL-R, and ADH1t-UNIVOL-F+ NEUT5homology-pDISR (**Supplementary Table 3**) as described previously[7]. Overlap extension PCR of the amplified products was performed and a final amplification of the sewn product was achieved using NEUT5homology-pDISF and NEUT5homology-pDISR primers to make the repair template. The transformation was performed using the previously mentioned CRISPR-Cas9 protocol, except the crNEUT5L-up_pDIS3 crRNA was used during the preparation of RNP assembly. Transformants were selected on YPD-200NAT. Correct genomic integration and gene reversion were confirmed by using primers NAT1INTF+NEUT5LAMPR and RBK1DETF+RBK1DETR **(Supplementary Table 3)** [6, 7].

Construction of *RBK1*, *HGT7*, or double overexpression strains

To construct overexpression strains *zcf13*Δ/Δ+Pr*TEF1-RBK1* or *zcf13*Δ/Δ+Pr*TEF1-HGT7,* the coding sequence of *RBK1* and *HGT7* was PCR amplified from SC5314 genomic DNA using primer pairs RBK1_ORF-F-SalI+ RBK1_ORF-R-MluI or HGT7_ORF-F-SalI+ HGT7_ORF-R-MluI, respectively. Plasmid pKE4 and PCR amplified products were then digested with restriction enzymes SalI+MluI and ligated to make pKE4-*RBK1* or -*HGT7* (**Supplementary Table 4**)*.* Part of *the* NEUT5 homology region and ADH1terminator were PCR amplified from pDIS3-tAHD1 as previously described. Plasmids pKE4-Pr*TEF1-RBK1* or -*HGT7* were amplified using primers PrTEF1OLF and tADH1OLR. The overlap extensions of the amplified products and subsequent steps were performed exactly as described above. Genotypes were confirmed by using primer pairs NAT1INTF+NEUT5LAMPR and either RBK1DETF+NAT1_INTR or HGT7DETF+NAT1_INTR.

The *RBK1* and *HGT7* double overexpression strain (*zcf13*Δ/Δ+Pr*TEF1-RBK1-HGT7*) was constructed using strain *zcf13*Δ/Δ+Pr*TEF1-HGT7.* Part of the *NEUT5* homology region, Pr*TEF1*-

RBK1, and the hygromycin resistance cassette were amplified from either pDIS3-tAHD1, pKE4-Pr*TEF1-RBK1,* or pHygR-FLP by using primer pairs NEUT5homology-pDISF+Nt5ADHup-UNIVOL-R, PrTEF1OLF+ADHterm-OL-R, PrHygR-OL-F+tHygR-OL-R, or NEUT5L_OL-F+ NEUT5homology-pDISR, respectively as described above. Transformants were selected on YPD-200NAT or YPD-600HygB medium. The genotype was confirmed using primers RBK1DETF+ADH13SEQR and NEUT5LAMPF+ CaHygB-SEQR.

RNA-Seq library preparation

For *C. albicans*, 1 µg RNA per sample was used for library preparation. Sequencing libraries were generated using NEBNext®Ultra™ RNA Library Prep Kit for Illumina® (New England Biolabs [NEB], Cat. No. E7530) following manufacturer's recommendations. Briefly, mRNA was purified from total RNA using poly-T oligo-attached magnetic beads. Fragmentation was carried out using divalent cations under elevated temperature in NEBNext First Strand Synthesis Reaction Buffer. First strand cDNA was synthesized using random hexamer primers and M- MuLV Reverse Transcriptase (RNase H-). Second strand cDNA synthesis was subsequently performed using DNA Polymerase I and RNase H. Remaining overhangs were converted into blunt ends via exonuclease/polymerase activities. After adenylation of 3' ends of DNA fragments, NEBNext Adaptor with hairpin loop structures were ligated to prepare for hybridization. Size-selected cDNA fragments of preferentially 150~200 bp in length were purified with AMPure XP system (Beckman Coulter, Cat. No. A63880). Then 3 µl USER™ enzyme (NEB, Cat No. M5505S) was used with size-selected, adaptor-ligated cDNA and PCR was performed with Phusion High-Fidelity DNA polymerase. Lastly, PCR products were purified (AMPure XP system) and library quality was assessed on the Agilent Bioanalyzer 2100 system.

For *S. aureus,* ribosomal RNA was removed from total RNA using Illumina Ribo-Zero Plus rRNA depletion Kit (Illumina, Cat. No. 20040526) according to the manufacturer's instructions, followed by ethanol precipitation. During the second strand cDNA synthesis, dUTPs were replaced with dTTPs in the reaction buffer. The directional library was ready after end repair, A-tailing, adapter

ligation, size selection, USER enzyme digestion, amplification, and purification. The rest of the sequencing pipeline was similar for both microbes. In brief, samples were clustered by a cBot Cluster Generation System using PE Cluster Kit cBot-HS according to the manufacturer's protocol and were sequenced by generating 150 bp paired-end reads using an Illumina platform.

RNA-Seq raw data processing

Raw data in FASTQ format were processed and trimmed by removing adapters, poly-N, and low-quality reads. At the same time, Q20, Q30, and GC content of the cleaned data were calculated and used for downstream analyses. The *C. albicans* SC5314 (C_albicans_SC5314_version_A22-s07-m01-r101_default_genomic.fasta) and *S. aureus* FPR3757 (000013465.ASM1346v1.dna.chromosome.Chromosome.fa.gz) reference genomes and gene model annotation files were downloaded from the Candida Genome Database or Ensemble Genomes, respectively. HISAT2 [8] or Bowtie2 [9] were used for *C. albicans* and *S. aureus,* respectively, to build a genome indices and perform alignments (**Supplementary Table 6**).The read numbers mapped to each gene were counted using FeatureCounts [10]. Gene expression levels were estimated by the expected number of Fragments Per Kilobase of transcript sequence per Million base pairs (FPKM) sequenced [11].

Homology search and alignment of ScGal2 orthologs

The *S. cerevisiae* Gal2 amino acid sequence was retrieved from the *Saccharomyces* Genome Database and a protein homology search conducted against the *C. albicans* SC5314 genome using NCBI BLAST. Clustal Omega was used to perform a multiple sequence alignment between ScGal2p and the homologous CaHgt7p and CaHgt8p amino acid sequences.

SUPPLEMENTARY TABLES

Strain	Organism	Parent	Relevant Genotype	Reference
DH5α	*E. coli*	--	Amp^S; cloning host	(12)
IM08B	E. coli	DC10B	Amp^S; SA08BΩPN25-hsdS (CC8-1) (SAUSA300_0406) of NRS384 integrated between the essQ and cspB genes	(13)
JE2	*S. aureus*	LAC	Erm^S, Cm^S	(14)
S. aureus(pDB22)	*S. aureus*	JE2	Erm^R, Cm^R; contains *agr* P3-GFP reporter plasmid pDB22	(2)
S. aureus(pOLux)	*S. aureus*	JE2	Erm^R, Cm^R; contains *agr* P3-*luxABCDE* reporter plasmid pOLux3	this study
hla::*bursa*	*S. aureus*	JE2	Erm^R; *hla*::*bursa*	(14)
hla::*bursa*-p*hla*	*S. aureus*	*hla*::*bursa*	Erm^R, Cm^R; contains plasmid pSK-*hla*	(2)
hla::*bursa*-p*hla*^{H35L}	*S. aureus*	*hla*::*bursa*	Erm^R, Cm^R; contains plasmid pSK-H35L	this study

Supplementary Table 1. Bacterial strains constructed or used in this study. Sensitivity (^{S}) or resistance (^{R}) to the following antibiotics is annotated: ampicillin (Amp), erythromycin (Erm), or chloramphenicol (Cm).

Strain name	Parent	Genotype	Reference
SC5314	--	wild-type, reference isolate	(15)
TF WT	SN152	*arg4Δ/arg4Δ leu2Δ/LEU2 his1Δ/HIS1 URA3/ura3Δ*::imm434 *IRO1/iro1Δ*::imm434	(16)
TF *zcf13Δ/Δ* (TF6)	SN152	*arg4Δ/arg4Δ zcf13Δ::LEU2 zcf13Δ::HIS1 URA3/ura3Δ*::imm434 *IRO1/iro1Δ*::imm434	(16)
zcf13-het	SC5314	*zcf13Δ:FRT / ZCF13 NEUT5L/NEUT5L*	this study
zcf13Δ/Δ	*zcf13*-het	*zcf13Δ:FRT zcf13Δ:FRT NEUT5L/NEUT5L*	this study
zcf13Δ/Δ+ZCF13	*zcf13Δ/Δ*	*zcf13Δ:FRT zcf13Δ:FRT neut5lΔ*:Pr*ZCF13-ZCF13*-t*ZCF13/NEUT5L*	this study
rbk1Δ/Δ	SC5314	*rbk1Δ:FRT+ rbk1Δ:FRT+ NEUT5L/NEUT5L*	this study
rbk1Δ/Δ+RBK1	*rbk1Δ/Δ*	*rbk1Δ:FRT+ rbk1Δ:FRT+ neut5lΔ*:Pr*RBK1-RBK1*-t*ADH1/NEUT5L*	this study
zcf13Δ/Δ+Pr*TEF1-RBK1*	*zcf13Δ/Δ*	*zcf13Δ:FRT+ zcf13Δ:FRT+ neut5lΔ*:Pr*TEF1-RBK1*-t*ADH1/NEUT5L*	this study
zcf13Δ/Δ-Pr*TEF1-HGT7*	*zcf13Δ/Δ*	*zcf13Δ*:FRT+ *zcf13Δ:FRT+ neut5lΔ*:Pr*TEF1-HGT7*-t*ADH1/NEUT5L*	this study
zcf13Δ/Δ-Pr*TEF1-HGT7-RBK1*	*zcf13Δ/Δ*	*zcf13Δ:FRT+ zcf13Δ:FRT+ neut5lΔ*:Pr*TEF1-HGT7*-t*ADH1 neut5lΔ*:Pr*TEF1-RBK1*-t*ADH1*	this study

Supplementary Table 2. *C. albicans* strains constructed or used in this study.

name	Sequence (5’ → 3’)[a]
**Primers**	
H35L-Superfi-F	ATGGCATGC***T***CAAAAAAGTATTTTATAG
H35L-Superfi-R	TACTTTTTTG***A***GCATGCCATTTTCTTTATC
hlaSeqF	GATATGTCTCAACTGCAATATTCTAAATTGACATA
hlaSeqR	ACATCATTTCTGAAGTTATCGGC
SynthGeneF	CGCAGTTACGGATCAGTCAC
SynthGeneRv4	GGTGCTGCCATGTTCTTTGCT
P3lux-F-NotI	TCAGCGGCCGCATTTTAACATAAAAAAATTTACAGTTAAGAATAAAAAACGACTAG
P3Lux-R-NcoI	TCACCATGGGATCTCTGTAATCTAGTTATATTAAAACATGCTAAAAGCAT
luxABCDE-Fv2-EcoRI	TCAGAATTCATTTTAACATAAAAAAATTTACAGTTAAGAATAAAAAACGACTAG
luxABCDE-R-NarI	TCAGGCGCCGATCACCGCGGCCATGAT
sarAP1-F-Acc65I	TCAGGTACCCTGATATTTTTGACTAAACCAAATGC
luxC-DET-R	GTCACGAATGTATGTCCTGC
ZCF13-FF-KpnI	TCAGGTACCAATCAAGCCTCCTGTACCACCACCA
ZCF13-FR-ApaI	TCAGGGCCCGCCTGGACTATTTGTCTTATCCATAATCGAT
ZCF13-RF-NotI	TCAGCGGCCGCGTGATGAAATTTTGGATCCTGTTGCTTG
ZCF13-RR-SacI	TCAGAGCTCGGCATGTTGTTGCTTTAGTGTCAGG
ZCF13-AMPF-SmaI	TCACCCGGGGACATCTCTCATTTGGTATAAATGATTGTCGG
ZCF13-AMPR-NotI	TCAGCGGCCGCTTTAGTGTCAGGTGTTAACACAACA
ZCF13-FLPINT-F	CAAGCCTCCTGTACCACCACCA
ZCF13-FLPINT-R	GCTTTAGTGTCAGGTGTTAACACAACACT
ZCF13-DET-F	CCACAACTGCAACAATCACAACAT
ZCF13-DET-R	CGATGATGGAGCTGTTTGATCAGAT
ZCF13-SEQ1-F	GTATCCCCCTCAACTAGCAGTTAG
ZCF13-SEQ2-F	CACCACCTTCTGTAACGACACCA
ZCF13-SEQ3-F	GCCAATTTCACTGATGCATTTGACATGA
ZCF13-SEQ4-F	CCGCACGTTTCAGAAGATCCC
ZCF13-SEQ5-F	GCCGCTAGTGATCAACTGTTTTTCC
Neut5homologyF	GCAGATATGAGATAAAAGTTTTAAAGGACAAGAAAAGG
RBK1CC9KO-F	ACAAAGTTTCTAGTATACTTAAAGTTGTGTTATTAAGTAGGGTTTTCCCAGTCACGACGT

RBK1CC9KO-R	ATAGTCAGGCAGAAGAGTTGTATTTAGAGAGGTATGACGGTGTGGAATTGTGAGCGGAT
RBK1DETF	GGTGATGATTCATTTGGTCAACAATTG
RBK1DETR	CGCCAGTAGAGTTTGTGACATTAC
RBK1INTF	GAGCTGAATTGGAAGCTGCTG
RBK1INTR	GTGCTGGTACTATGATTGCTGC
FLPINTF	CGCGCGTAATACGACTCACT
FLPINTR	CAAGCGCGCAATTAACCCTC
RBK1_Pr_OL-F	CCTCGAGGTCGACGGTATCGGTAGAGATGTTGAACCGAACCTAATGG
RBK1_ORF-OL-R	ATTTGCTTAGCATGCACGCGCTACAAAATTTCCATTACTTCTTCATAACT
NEUT5homology-pDISF	AGGAGGCTCCCCAAAGATTTTATCA
NEUT5homology-pDISR	TAACACACTGAATTCTACATCGAACAAGAGAAA
Nt5ADHup-UNIVOL-R	CGATACCGTCGACCTCGAGG
ADH1t-UNIVOL-F	CGCGTGCATGCTAAGCAAAT
NAT1INTF	CCCAGATGCGAAGTTAAGTGCG
NEUT5LAMPR	GGAATTTCTAGTCACTTGACACGACC
RBK1_ORF-F-SalI	TCA<u>GTCGAC</u>ATGTCATCCTCCAATCCATCAATTACTAT
RBK1_ORF-R-MluI	TCA<u>ACGCGT</u>CTACAAAATTTCCATTACTTCTTCATAACT
PrTEF1OLF	CCTCGAGGTCGACGGTATCGCTGCAAATCTGTTTGCTGATGGACA
tADH1OLR	ATTTGCTTAGCATGCACGCG
HGT7_ORF-F-SalI	TCA<u>GTCGAC</u>ATGTCTCAAGACAACGTCTCATCA
HGT7_ORF-R-MluI	TCA<u>ACGCGT</u>TTAAACGTGTTCCTCTTCTGGTTT
HGT7DETF	ACAGCTGAGGCTGTAAATAATGAAA
NAT1_INTR	CCATCCAAAGCTTCAATAGCTTCAGC
ADHterm-OL-R	CGAAAACTTGAAACTTGAAAACACCG
PrHygR-OL-F	CGGTGTTTTCAAGTTTCAAGTTTTCGCCATCATAAAATGTCGAGCGTCAAAAC
tHygR-OL-R	CTGCAGAGGACCACCTTTGATT
NEUT5L_OL-F	AATCAAAGGTGGTCCTCTGCAGCAGTGTCGACGTTTAAACGAGCTC
ADH13SEQR	ATATCGCACTCACGTAAACAC
NEUT5LAMPF	GCTGAATCACTTGATAGGATTTAGTTCCATTATGG
CaHygB-SEQR	CCAAATGTCTAACTTCTGGACAATCTTCA
QPCR_RBK1_F	CAAACTTCAGGAGTTGCGGTG

QPCR_RBK1_R	GGTGGTGGTGATGCTTCCAG
QPCR_ZCF13_F	CACCGCAAGAAAGCGAATCA
QPCR_ZCF13_R	GTTGCCATTCATTACCCGTTGA
QPCR_HGT7_F	ACAGCTGAGGCTGTAAATAATGAAA
QPCR_HGT7_R	ACCACCAAAGGCAATAAGGAAAC
QPCR_HGT8_F	AAGAGTCTTTGTGGGTGCCA
QPCR_HGT8_R	ACACCAATAATGATGGACGTTTGG
CaACT1-F-qPCR	TTGGATTCTGGTGATGGTGTTA
CaACT1-R-qPCR	TCAAGTCTCTACCAGCCAAATC
**crRNAs**	
crRBK1up	AGATGTTGAACCGAACCTAA
crRBK1down	GTTGTTGTCGCTAGTGTTTA
crNEUT5L-up_pDIS3	GCTCGGAGGAGGCTCCCCAA

Supplementary Table 3: Oligonucleotides and crRNAs used in this work. Point mutations are bolded and italicized. Restriction enzyme sites are underlined.

Plasmid	Relevant characteristics	Reference
pDB22	*agr* P3-GFPmut2 reporter; Erm^{R}	(17)
pMK4	high copy number plasmid; Amp^{R}, Cm^{R}	(18)
pSK5630	low copy number plasmid; Amp^{R}, Cm^{R}	(19)
pSK-hla	pSK5630 containing Pr*hla-hla* amplified from JE2	(2)
pSK-H35L	mutagenesis of pSK-hla using primers H35L-SuperFi-F and H35L-SuperFi-R	this study
pMV306G13+Lux	contains *luxABCDE* operon from *Mycobacterium marinum* and *Photorhabdus luminescens*; Kan^{R}	(20)
pOLux1	NotI and NcoI digested pMV306G13+Lux containing *agr* P3 promoter amplified from synthetic DNA *agr*P3 with primers SynthGeneF and SynthGeneRv4	this study
pOLux2	pMK4 containing *agr P3-luxABCDE* amplified from pOLux1 using primers luxABCDE-Fv2-EcoRI + luxABCDE-R-NarI	this study
pOLux3	Acc65I-digested pOLux2 containing the *sarA* P1 promoter amplified from synthetic DNA *sarA*P1 using primers SynthGeneF and SynthGeneRv4	this study
pBSS2	*SAT1*-flipper plasmid; NAT^{R}	(3)
pBSS2-ZCF13-F	plasmid pBSS2 containing 5’ flanking region of ZCF13 amplified from SC5314 gDNA using primers ZCF13-FF-KpnI and ZCF13-FR-ApaI	this study
pBSS2-ZCF13-FR	plasmid pBSS2-ZCF13-F 3’ flanking region of ZCF13 amplified from SC5314 gDNA using primers ZCF13-RF-NotI and ZCF13-RR-SacI	this study
pDUP3	contains *NEUT5L* homology regions; NAT^{R}	(21)
pDUP3-ZCF13	pDUP3 containing *C. albicans ZCF13* locus between SmaI and NotI restriction sites	this study
CaHygB-flipper	*C. albicans* optimized HygB-flipper plasmid; HygB^{R}	(7)
pDIS3-tADH1	contains *NEUT5L* homology regions and *C. albicans ADH1* terminator; NAT^{R}	(7)
pKE4	contains *C. albicans TEF1* promoter, *ADH1* terminator, and *URA3* marker	(22)
pKE4-RBK1	pKE4 containing *C. albicans* RBK1 open reading frame between SalI and MluI restriction sites	this study
pKE4-HGT7	pKE4 containing *C. albicans* HGT7 open reading frame between SalI and MluI restriction sites	this study

Supplementary Table 4. Plasmids constructed or used in this study. Sensitivity (^{S}) or resistance (^{R}) to the following antimicrobials is annotated: ampicillin (Amp), erythromycin (Erm), chloramphenicol (Cm), kanamycin (Kan), nourseothricin (NAT), hygromycin B (HygB).

Synthetic DNA	Sequence 5'→3'
*sarA*P1	CGCAGTTACGGATCAGTCACGGTACCCTGATATTTTTGACTAAACCAAATGCTAACCCAGAA ATACAATCACTGTGTCTAATGAATAATTTGTTTTATAAACACTTTTTTGTTTACTTCTCATTTTT AATTAGTTATAATTAACTAAATAATAGAGCATTAAATATATTTAATAAAACTTATTTAATGCAAA ATTATGACTAACATATCTATAATAAATAAAGATTAGATATCAATATATTATCGGGCAAATGTAT CGAGCAAGATGCATCGGTACCAGCAAAGAACATGGCAGCACC
*agr*P3	CGCAGTTACGGATCAGTCACGCGGCCGCATTTTAACATAAAAAAATTTACAGTTAAGAATAA AAAACGACTAGTTAAGAAAAATTGGAAAATAAATGCTTTTAGCATGTTTTAATATAACTAGATT CCATGGAGCAAAGAACATGGCAGCACC

Supplementary Table 5. Synthetic DNA sequences used in this study.

Sample	Description	Total Reads Obtained	# Reads Mapped to Ref Genome	% Reads Mapped to Ref Genome
***C. albicans* samples**				
TF_WT_1	TF library control strain (rep 1)	40,668,310	39,306,969	96.7%
TF_WT_2	TF library control strain (rep 2)	55,404,172	53,547,217	96.7%
TF_WT_3	TF library control strain (rep 3)	39,481,300	38,322,397	97.1%
TF_Z_1	TF *zcf13*Δ/Δ (rep 1)	41,994,578	40,791,150	97.1%
TF_Z_2	TF *zcf13*Δ/Δ (rep 2)	49,034,592	47,232,306	96.3%
TF_Z_3	TF *zcf13*Δ/Δ (rep 3)	44,139,832	42,910,918	97.2%
S. aureus samples				
SCTRL_1	untreated control	16,651,994	16,588,211	99.6%
SCTRL_2	untreated control	18,145,960	18,075,554	99.6%
SCTRL_3	untreated control	19,531,768	19,458,825	99.6%
SCTRL_4	untreated control	17,597,256	17,530,560	99.6%
SRIBH_1	ribose treated	14,238,240	14,180,783	99.6%
SRIBH_2	ribose treated	15,223,534	15,156,847	99.5%
SRIBH_3	ribose treated	16,051,650	15,985,920	99.6%
SRIBH_4	ribose treated	13,630,960	13,579,086	99.6%

Supplementary Table 6. RNA-seq mapping statistics in each sample and sample key.

SUPPLEMENTARY FIGURES

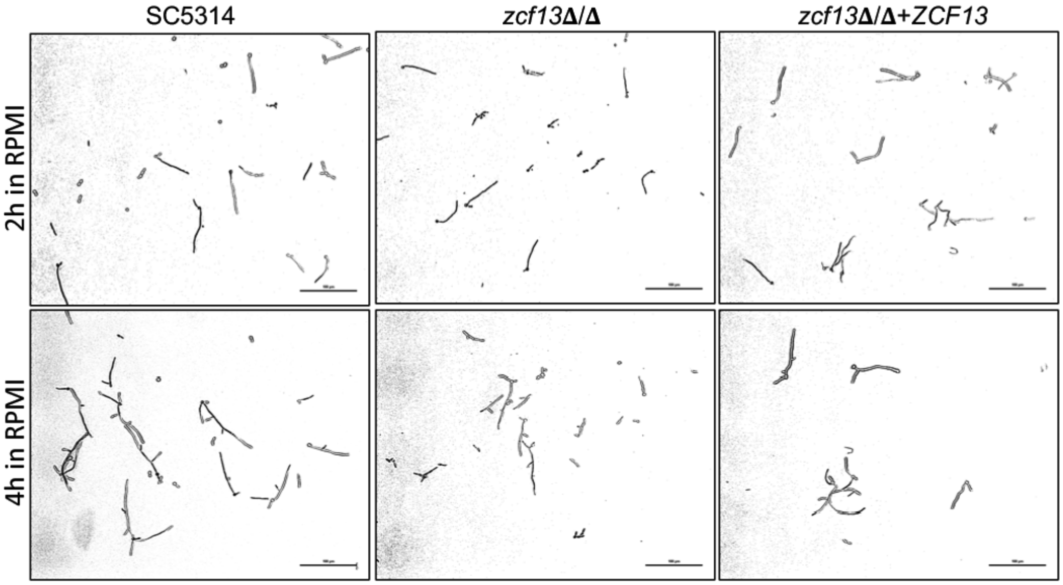

Supplementary Fig 1: *ZCF13* deletion does not impact hyphal growth. SC5314, *zcf13*Δ/Δ, and *zcf13*Δ/Δ+*ZCF13* cells were inoculated in RPMI containing 165 mM MOPS, pH 7.0 at 37ºC, and hyphal formation was monitored at 2 h and 4 h. Images were digitally captured by standard light microscopy. Scale bar is 100 μm. Images are representative of duplicate experiments.

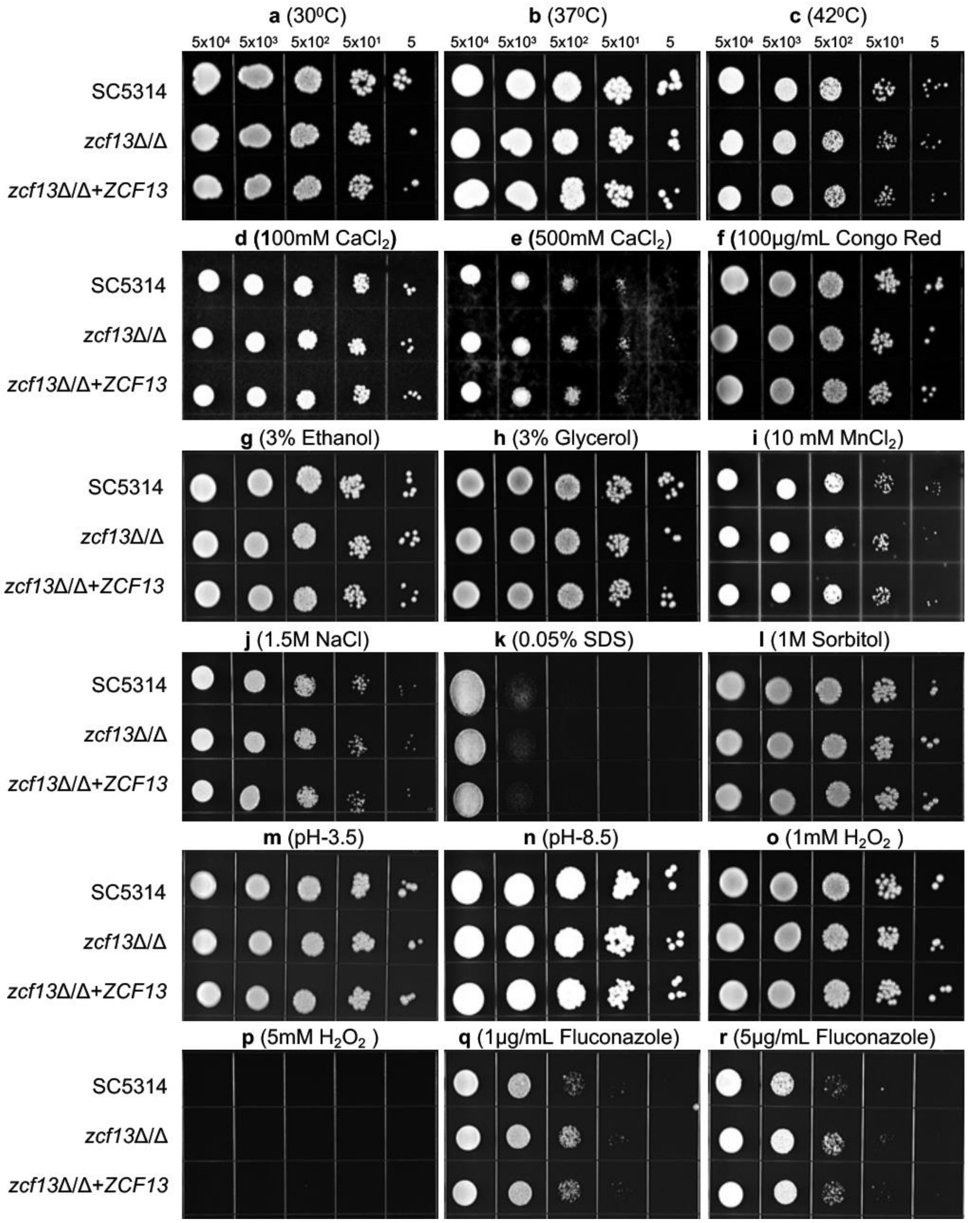

Supplementary Fig. 2: *ZCF13* deletion does not increase susceptibility to a variety of stressors. The indicated number of SC5314, *zcf13*Δ/Δ, *zcf13*Δ/Δ+*ZCF13* cells were spotted on YPD medium containing various stressors or incubated under varied temperature as indicated: **a**, **b, c** temperature, **d, e** CaCl2, **f** Congo Red, **g** ethanol, **h** glycerol, **i** MnCl2, **j** NaCl, **k** SDS, **l** sorbitol, **m, n** pH, **o, p** H2O2, **q, r** fluconazole. Images were digitally captured. Images are representative of duplicate experiments.

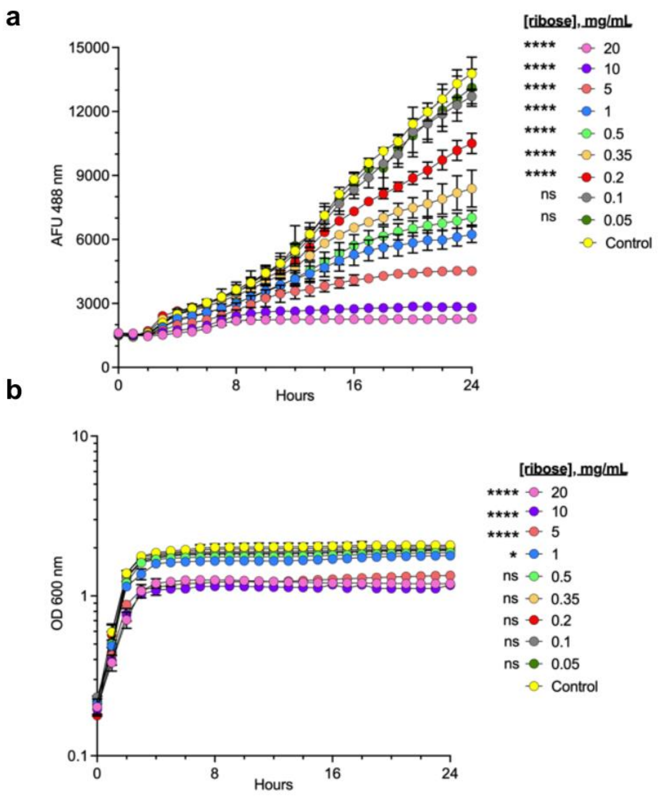

Supplementary Fig. 3: Ribose dose-dependently inhibits *agr* activation and growth in *S. aureus*. *S. aureus* was grown in TSBg or that supplemented with increasing concentrations of ribose (see key) and **a** fluorescence (488 nm) and **b** growth (OD600 nm) were kinetically monitored. All experiments were repeated in biological triplicate and shown as mean ± SEM. Significance was assessed using a one–way ANOVA and Dunnet's test at the endpoint. *, p=0.0273; ****, p < 0.0001.

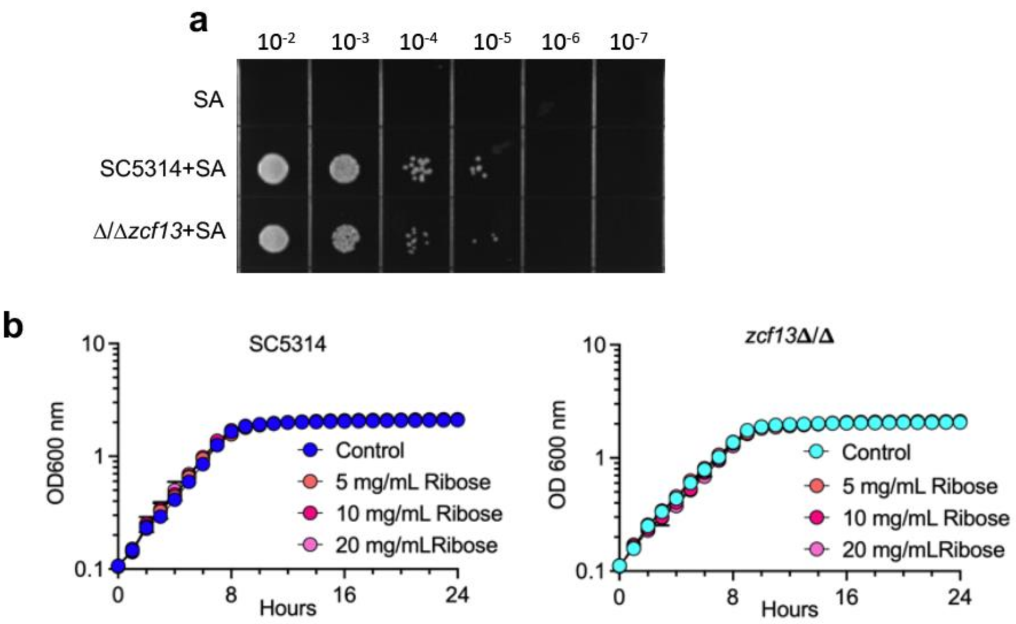

Supplementary Fig. 4: Ribose is not growth inhibitory for *C. albicans*. a. SC5314, *zcf13*Δ/Δ and *zcf13*Δ/Δ+ZCF13 were cocultured with S. aureus(pDB22) in TSBg supplemented with 0.5 mg/mL ribose for 16 h. Indicated dilutions were made, spotted onto YPD containing 50 µg/mL chloramphenicol, and cell growth monitored at 24 h. Images are representative of 3 independent experiments. **b** SC5314 and *zcf13*Δ/Δ were grown in TSBg supplemented with indicated concentrations of ribose for 16 h and growth temporally monitored at OD600 nm using a spectrophotometer. Data is depicted as the mean ± SEM of 3 independent experiments.

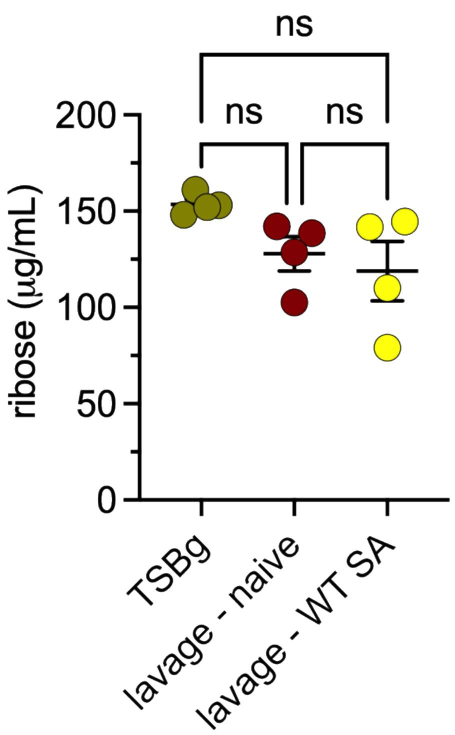

Supplementary Fig. 5: In vitro ribose levels mirror those of the murine peritoneal environment. Fresh TSBg (brown) or peritoneal lavage fluid obtained from uninfected (naïve, maroon) or *S. aureus* (yellow) challenged mice (n=4 per group) were obtained at 8 h p.i. The concentration of ribose measured by LC-MS. Significance was assessed using one-way ANOVA and Tukey's test. n.s., not significant.

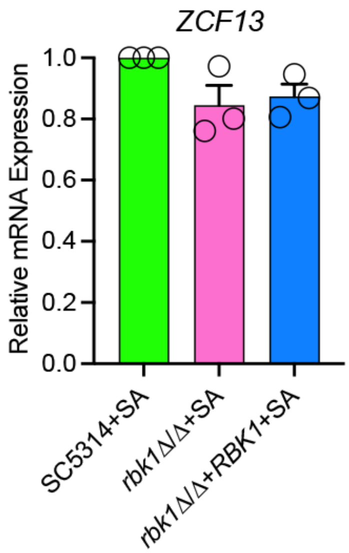

Supplementary Fig. 6: *RBK1* deletion does not impact *ZCF13* expression. SC5314 (green), *rbk1*Δ/Δ (pink), *rbk1*Δ/Δ and *rbk1*Δ/Δ+*RBK1* (blue) were co-cultured with *S. aureus*(pDB22) in TSBg. *ZCF13* expression was measured by quantitative real-time PCR and normalized by $2^{-\Delta\Delta Ct}$ to *ACT1* and SC5314+SA using gene-specific primers**.** Data is depicted as the mean + SEM from 3 independent experiments.

```
name                              sequence                          position

ScGal2p     MAVEENNMPVVSQQPQAGEDVISSLSKDSHLSAQSQKYSN--DELKAGESGSEGSQSVPI      58
CaHgt7p     ----------MSQ------------DNVSSTSTA----EAVNNEIKV-KDEFPQEEQAHT      33
CaHgt8p     ----------MSS------------TNSTENHAVEEKYEDPQQQQQ-QQQQQQQQQKD      37
                      :*.             : :   :     .   :: :  :.    .:.

ScGal2p     EIPKKPMSEYVTVSLLCLCVAFGGFMFGWDTGTISGFVVQTDFLRRFGMKHKDGTHYLSN      118
CaHgt7p     SLEDKPVSAYIGIIIMCFLIAFGGFVFGFDTGTISGFINMSDFLERFGGTKADGTLYFSN      93
CaHgt8p     ALAKKPMSAYIGISIMCVLIAFGGFVFGFDTGTISGFINMSDFLERFGGTRADGTLYFSN      97
             : .**:* *: : ::*. :*****:**:********:  :***.*** .: *** *:**

ScGal2p     VRTGLIVAIFNIGCAFGGIILSKGGDMYGRKKGLSIVVSVYIVGIIIQIASINKWYQYFI      178
CaHgt7p     VRTGLMIGLFNAGCAIGALFLSKVGDMYGRRVGIMTAMIVYIVGIIVQIASQHAWYQVMI      153
CaHgt8p     VRTGLLIGLFNVGCAIGALFLSKVGDMYGRRVGIMTAMIIYIVGIIVQIASQHAWYQVMI      157
            *****::.:** ***:*.::*** ******: *:  .: :******:**** : *** :*

ScGal2p     GRIISGLGVGGIAVLCPMLISEIAPKHLRGTLVSCYQLMITAGIFLGYCTNYGTKSYSNS      238
CaHgt7p     GRIITGLAVGMLSVLCPLFISEVSPKHLRGTLVCCFQLMITLGIFLGYCTTYGTKSYSDS      213
CaHgt8p     GRIITGLAVGTLSVLCPLFISEVSPKHLRGTLVCCFQLMITLGIFLGYCTTYGTKTYSDS      217
            ****:**.** ::****::***::*********.*:***** ********.****:**:*

ScGal2p     VQWRVPLGLCFAWSLFMIGALTLVPESPRYLCEVNKVEDAKRSIAKSNKVSPEDPAVQAE      298
CaHgt7p     RQWRIPLGLCFAWALCLVAGMVRMPESPRYLVGKDRIEDAKMSLAKTNKVSPEDPALYRE      273
CaHgt8p     RQWRIPLGLCFAWALCLLGGMVRMPESPRYLVGNDRIEDAKISLAKTNKVSPEDPALYRE      277
             ***:********:* ::..:. :*******   :::**** *:**:*********:  *

ScGal2p     LDLIMAGIEAEKLAGNASWGELFSTKTKVFQRLLMGVFVQMFQQLTGNNYFFYYGTVIFK      358
CaHgt7p     LQLIQAGVERERLAGKASWGTLFNGKPRIFERVIVGVMLQALQQLTGDNYFFYYSTTIFK      333
CaHgt8p     LQLIQAGVERERLAGSASWKSLITGKPRILERVFVGAMLQSLQQLTGDNYFFYYSTTIFK      337
            *:** **:* *:***.***  *:. * ::::*:::*.::* :*****:******.*.***

ScGal2p     SVGLDDSFETSIVIGVVNFASTFFSLWTVENLGHRKCLLLGAATMMACMVIYASVGVTRL      418
CaHgt7p     SVGMNDSFETSIIIGVINFASTFVGIYAIERMGRRLCLLTGSVAMSICFLIYSLVGTQHL      393
CaHgt8p     SVGLNDSFQTSIIIGVINFASTFVGIYAIEKLGRRHCLLIGSVAMSICFLIYSLIGTQHL      397
            ***::***:***:***:******..::::*.:*:* *** *:.:*  *::**: :*. :*

ScGal2p     YPHGKSQPSSKGAGNCMIVFTCFYIFCYATTWAPVAWVITAESFPLRVKSKCMALASASN      478
CaHgt7p     YIDKPGGASRKPDGDAMIFMTSLYVFFFASTWAGGVYSIISELYPLKVRSKAMGLANASN      453
CaHgt8p     YIDKPGGASRKPDGDAMIFITALYVFFFASTWAGGVYSIMSELYPLKVRSKAMGIASASN      457
            * .  .  * *  *:.**.:*.:*:* :*:***  .: * :* :**:*:**.*.:*.***

ScGal2p     WVWGFLIAFFTPFITSAINFYYGYVFMGCLVAMFFYVFFFVPETKGLSLEEIQELWEEGV      538
CaHgt7p     WTWGFLISFFTSFITDAIHFYYGFVFMGCLVFSIFFVYFMVYETKGLTLEEIDELYSTKV      513
CaHgt8p     WLWGFLISFFTSFITDAIHFYYGFVFMGCLVFSIFFVYFMVYETKGLTLEEIDELYSTKV      517
            * *****:*** ***.**:****:*******  :*:*:*:* *****:****:**:.  *

ScGal2p     LPWKSEGWIPSSRRGNNYDLEDLQHDDKPWYKAMLE*      574
CaHgt7p     LPWKSAGWVPPSEEEMATSTG-YAGDAKPEEEHV*--      546
CaHgt8p     VPWKSAGWVPPSEEEMATSTG-YAGDAKPTEEHV*--      550
            :**** **:* *..    .      * **  : :
```

Supplementary Fig. 7: ***C. albicans*** **Hgt7p and Hgt8p are close orthologues to the** ***S. cerevisiae*** **low affinity ribose importer Gal2p.** A multiple sequence alignment was performed using Clustal Omega. “*” indicate exact matches; “:” or “.” indicate matches to amino acids with strongly or weakly similar properties, respectively.

SUPPLEMENTARY REFERENCES

1. Todd, O.A., Noverr, M.C. & Peters, B.M. *Candida albicans* Impacts *Staphylococcus aureus* Alpha-Toxin Production via Extracellular Alkalinization. *mSphere* **4** (2019).
2. Todd, O.A. et al. *Candida albicans* Augments *Staphylococcus aureus* Virulence by Engaging the Staphylococcal *agr* Quorum Sensing System. *mBio* **10** (2019).
3. Reuss, O., Vik, A., Kolter, R. & Morschhauser, J. The *SAT1* flipper, an optimized tool for gene disruption in *Candida albicans*. *Gene* **341**, 119-127 (2004).
4. Gietz, D., St Jean, A., Woods, R.A. & Schiestl, R.H. Improved method for high efficiency transformation of intact yeast cells. *Nucleic Acids Res* **20**, 1425 (1992).
5. Znaidi, S. et al. The zinc cluster transcription factor Tac1p regulates *PDR16* expression in *Candida albicans. Mol Microbiol* **66**, 440-452 (2007).
6. Miao, J. et al. Glycogen Metabolism in *Candida albicans* Impacts Fitness and Virulence during Vulvovaginal and Invasive Candidiasis. *mBio* **14**, e0004623 (2023).
7. Liu, J. et al. Rapid Hypothesis Testing in *Candida albicans* Clinical Isolates Using a Cloning-Free, Modular, and Recyclable System for CRISPR-Cas9 Mediated Mutant and Revertant Construction. *Microbiol Spectr* **10**, e0263021 (2022).
8. Kim, D., Langmead, B. & Salzberg, S.L. HISAT: a fast spliced aligner with low memory requirements. *Nat Methods* **12**, 357-360 (2015).
9. Langmead, B. & Salzberg, S.L. Fast gapped-read alignment with Bowtie 2. *Nat Methods* **9**, 357-359 (2012).
10. Liao, Y., Smyth, G.K. & Shi, W. featureCounts: an efficient general purpose program for assigning sequence reads to genomic features. *Bioinformatics* **30**, 923-930 (2014).
11. Trapnell, C. et al. Transcript assembly and quantification by RNA-Seq reveals unannotated transcripts and isoform switching during cell differentiation. *Nat Biotechnol* **28**, 511-515 (2010).
12. Chen, J., Li, Y., Zhang, K. & Wang, H. Whole-Genome Sequence of Phage-Resistant Strain Escherichia coli DH5alpha. *Genome Announc* **6** (2018).
13. Monk, I.R., Tree, J.J., Howden, B.P., Stinear, T.P. & Foster, T.J. Complete Bypass of Restriction Systems for Major *Staphylococcus aureus* Lineages. *mBio* **6**, e00308-00315 (2015).
14. Fey, P.D. et al. A genetic resource for rapid and comprehensive phenotype screening of nonessential Staphylococcus aureus genes. *mBio* **4**, e00537-00512 (2013).
15. Gillum, A.M., Tsay, E.Y. & Kirsch, D.R. Isolation of the *Candida albicans* gene for orotidine-5'-phosphate decarboxylase by complementation of *S. cerevisiae ura3* and *E. coli pyrF* mutations. *Mol Gen Genet* **198**, 179-182 (1984).
16. Homann, O.R., Dea, J., Noble, S.M. & Johnson, A.D. A phenotypic profile of the *Candida albicans* regulatory network. *PLoS Genet* **5**, e1000783 (2009).
17. Yarwood, J.M., Bartels, D.J., Volper, E.M. & Greenberg, E.P. Quorum sensing in *Staphylococcus aureus* biofilms. *J Bacteriol* **186**, 1838-1850 (2004).
18. Sullivan, M.A., Yasbin, R.E. & Young, F.E. New shuttle vectors for *Bacillus subtilis* and *Escherichia coli* which allow rapid detection of inserted fragments. *Gene* **29**, 21-26 (1984).
19. Grkovic, S., Brown, M.H., Hardie, K.M., Firth, N. & Skurray, R.A. Stable low-copy-number *Staphylococcus aureus* shuttle vectors. *Microbiology (Reading)* **149**, 785-794 (2003).

20. Andreu, N. et al. Optimisation of bioluminescent reporters for use with mycobacteria. *PLoS One* **5**, e10777 (2010).
21. Gerami-Nejad, M., Zacchi, L.F., McClellan, M., Matter, K. & Berman, J. Shuttle vectors for facile gap repair cloning and integration into a neutral locus in *Candida albicans*. *Microbiology (Reading)* **159**, 565-579 (2013).
22. Willems, H.M.E. et al. Overexpression of *Candida albicans* Secreted Aspartyl Proteinase 2 or 5 Is Not Sufficient for Exacerbation of Immunopathology in a Murine Model of Vaginitis. *Infect Immun* **85** (2017).
