# Supplementary material for: A fungal metabolic regulator underlies infectious synergism during Candida albicans-Staphylococcus aureus intra-abdominal co-infection
Source: Nat Commun. 2024 Jul 9;15:5746. doi: 10.1038/s41467-024-50058-w (PMC11233573; doi:10.1038/s41467-024-50058-w)
Supplement: Supplementary file 3 — Description of Additional Supplementary Files [file 41467_2024_50058_MOESM3_ESM.pdf]

Description of Additional Supplementary Files

Supplementary Data Legends:

Supplementary Data 1. Fold-change fluorescence and z-score values from agr P3 reporter screen with C. albicans transcription factor mutants.

Supplementary Data 2. RNA-Seq dataset of TF WT vs. TF zcf13Δ/Δ.

Supplementary Data 3. Kinetic OD600 nm reads from Biolog carbon source plates with SC5314, zcf13Δ/Δ, and zcf13Δ/Δ+ZCF13.

Supplementary Data 4. RNA-Seq dataset of S. aureus grown in the presence or absence of ribose.

Supplementary Data 5. Gene expression dataset of S. aureus grown in the presence or absence of ribose used to construct volcano plot.
